# Experimental Study of a Reference Model Vertical-Axis Cross-Flow Turbine

**DOI:** 10.1371/journal.pone.0163799

**Published:** 2016-09-29

**Authors:** Peter Bachant, Martin Wosnik, Budi Gunawan, Vincent S. Neary

**Affiliations:** 1 Center for Ocean Renewable Energy, University of New Hampshire, Durham, NH, 03824, United States of America; 2 Water Power Technologies, Sandia National Laboratories, P.O. Box 5800, Albuquerque, New Mexico 87185-MS1124, United States of America; Seoul National University, KOREA

## Abstract

The mechanical power, total rotor drag, and near-wake velocity of a 1:6 scale model (1.075 m diameter) of the US Department of Energy’s Reference Model vertical-axis cross-flow turbine were measured experimentally in a towing tank, to provide a comprehensive open dataset for validating numerical models. Performance was measured for a range of tip speed ratios and at multiple Reynolds numbers by varying the rotor’s angular velocity and tow carriage speed, respectively. A peak power coefficient *C*_*P*_ = 0.37 and rotor drag coefficient *C*_*D*_ = 0.84 were observed at a tip speed ratio *λ*_0_ = 3.1. A regime of weak linear *Re*-dependence of the power coefficient was observed above a turbine diameter Reynolds number *Re*_*D*_ ≈ 10^6^. The effects of support strut drag on turbine performance were investigated by covering the rotor’s NACA 0021 struts with cylinders. As expected, this modification drastically reduced the rotor power coefficient. Strut drag losses were also measured for the NACA 0021 and cylindrical configurations with the rotor blades removed. For λ = λ_0_, wake velocity was measured at 1 m (*x*/*D* = 0.93) downstream. Mean velocity, turbulence kinetic energy, and mean kinetic energy transport were compared with results from a high solidity turbine acquired with the same test apparatus. Like the high solidity case, mean vertical advection was calculated to be the largest contributor to near-wake recovery. However, overall, lower levels of streamwise wake recovery were calculated for the RM2 case—a consequence of both the relatively low solidity and tapered blades reducing blade tip vortex shedding—responsible for mean vertical advection—and lower levels of turbulence caused by higher operating tip speed ratio and therefore reduced dynamic stall. Datasets, code for processing and visualization, and a CAD model of the turbine have been made publicly available.

## Introduction

The Reference Model Project (RMP), sponsored by the US Department of Energy (DOE), produced six marine hydrokinetic (MHK) technology point designs as reference models (RMs) to serve as non-proprietary test articles for open research development, and to benchmark performance and costs for technology developers [1, 2]. Open-source RMP products, along with supporting documentation, are available at the RMP website http://energy.sandia.gov/rmp to facilitate their use in future R&D studies by industry, academia, and national laboratories. These products include: Technical specifications and computer-aided design (CAD) files for each RM device to allow exact replication for physical and numerical modeling studies; resource site information used to design each RM device; and references to physical modeling data sets that can be used to validate numerical modeling design and analysis tools.

Reference Model 2 (RM2) is a dual-rotor, vertical-axis cross-flow hydrokinetic (river) turbine that was designed to operate in a reach of the lower Mississippi River near Baton Rouge, Louisiana [3, 4], but could also be deployed in a tidal environment. The rotor has three tapered blades and a relatively low solidity *Nc*/(*πD*), where *N* is the number of blades, *c* is the chord length, and *D* is the rotor diameter, or chord-to-radius ratio *c*/*R* ≈ 0.1. A preliminary analysis with Sandia’s CACTUS vortex line numerical model [5] predicted an individual rotor’s full-scale performance coefficient to be $C_{P}\equiv P/(\frac{1}{2}\rho AU_{\infty }^{3})=0.47$—where *P* is the mechanical power output, *ρ* is the fluid density, *A* is the rotor frontal area, and *U*_∞_ is the free stream velocity—at 1 m/s flow speed and a tip speed ratio $\lambda \equiv \frac{\omega R}{U_{\infty }}=3.15$ [3], where *ω* and *R* are the rotor’s angular velocity and radius, respectively. Note that the RM2 rotor solidity would be considered moderate to high for a wind turbine, whose *c*/*R* values typically range from 0.05 to 0.09, but MHK rotors are typically higher solidity since they must withstand approximately an order of magnitude higher torque in typical flows, necessitating relatively larger blades to meet strength and fatigue life requirements.

An initial experimental measurement campaign with a 1:15 scale RM2 rotor conducted at the Saint Anthony Falls Laboratory (SAFL) at the University of Minnesota resulted in maximum power coefficient of approximately 5% at λ = 2.2 [6]. This discrepancy between numerical and physical model performance prompted the experimental measurements presented here, namely due to the low Reynolds number—*Re*_*D*_ = *U*_∞_ *D*/*ν*, where *U*_∞_ is the free stream velocity, *D* is the rotor diameter, and *ν* is the fluid kinematic viscosity—of the 1:15 scale tests, which were performed at *Re*_*D*_ ∼ 10^5^.

The effect of Reynolds number on average power output was shown to be significant on the 2 m Sandia Research Darrieus turbine in wind tunnel testing [7]: The maximum power coefficient, *C*_*P*_max__, increased with Reynolds number, *Re*_*c*_ = λ*U*_∞_ *c*/*ν* (based on blade chord rather than diameter, though these are approximately proportional since optimal tip speed ratio correlates inversely with solidity and therefore chord length), along with a shift of the location of *C*_*P*_max__ toward lower tip speed ratios due to delayed blade stall. The effects of Reynolds number were quite dramatic over a relatively small range of *Re*_*c*_ ≈ 1.1 × 10^5^–2.9 × 10^5^. More recently, Bachant and Wosnik [8, 9] showed that performance and near-wake characteristics of a high solidity cross-flow turbine became Reynolds number independent at *Re*_*D*_ ≈ 10^6^ or *Re*_*c*_ ≈ 2 × 10^5^.

As part of the engineering process it is generally less expensive to assess designs via numerical rather than physical models. However, it is important—especially when dealing with high Reynolds number fluid dynamics where the “exact” physics cannot be resolved with even the most advanced computers—that numerical models be validated against experimental data. It is uncertain whether numerical models validated with physical model data obtained at low Reynolds numbers should be considered validated at all, since the scale at which the model will be applied for real world design problems is orders of magnitude larger. One way to overcome this uncertainty is to show that the scaled physical model test has become Reynolds number independent, so validation efforts are relevant at full-scale, which was the strategy employed here.

The main objective of the present study was to acquire a new experimental dataset for the RM2 turbine at sufficiently high Reynolds numbers to be relevant to full-scale physical and numerical modeling. It was hypothesized that the parasitic losses from rotor blade support struts could play an important role in overall turbine performance, especially for a lower solidity turbine that operates at higher tip speed ratios. Thus, the parasitic torque from strut drag was measured without blades, then deliberately and dramatically increased in the physical model to provide data to investigate its importance. The velocity field in the near-wake of the turbine was then measured to compare with measurements from a higher solidity rotor in the same experimental setup [10], and to provide validation data for numerical models that predict wake flows, which determine turbine–turbine interaction and optimal spacing for turbine arrays. This dataset, along with the code for processing and visualization, has been made publicly available [11].

### Survey of Validation Data and Usage

A selection of measured performance data in the literature and its usage in numerical model validation is presented in Table 1. Turbine diameter Reynolds numbers spanned from small laboratory scale (*Re*_*D*_ ∼ 10^5^) all the way to full scale (*Re*_*D*_ ∼ 10^7^). Individual blade forces were only measured in two of the experiments—Strickland *et al.* [12] and Laneville and Vitecoq [13]. There has been nearly equal attention given to the eggbeater-shaped Darrieus rotor and the straight-bladed H-rotor. The performance of a large scale H-rotor with tapered blades, the VAWT 850 [14], has also been measured.

**Table 1:** Performance validation data. Selected measured performance data and its usage for numerical model validation. Note that individual blade forces were measured in the Strickland *et al.* and Laneville and Vitecoq experiments.

Name	Rotor type	*N*	*c*/*R*	*Re*_*D*_	Used in
Sandia 2 m [7]	Darrieus	2–3	0.06–0.09	∼10^6^	[15, 16]
Sandia 5 m [17]	Darrieus	3	0.08	∼10^6^	[16, 18]
Sandia 17 m [19]	Darrieus	2	0.06	∼10^7^	[16, 20, 21]
Sandia 34 m [22]	Darrieus	2	0.05	∼10^7^	[5, 16, 23]
Strickland *et al.* [12]	H	1–3	0.15	∼10^5^	[24, 25]
Laneville and Vitecoq [13]	H	2	0.13	∼10^6^	[26]
Howell *et al.* [27]	H	2–3	0.33	∼10^5^	[28]
Mertens [29]	H	2	0.21	∼10^5^	[21]
VAWT 850 [14]	Tapered H	2	0.05	∼10^7^	[5]
UNH-RVAT [30]	H	3	0.28	∼10^6^	[31]
RM2 (present)	Tapered H	3	0.07–0.12	∼10^6^	

In general, there have been more experiments done with low *c*/*R* rotors. These rotors are easier to model, since unsteady dynamic effects are less influential on the overall performance [12]. This is apparent when examining the effectiveness of numerical models that rely on static foil coefficient input data, e.g., streamtube and vortex models, which are most applicable for *c*/*R* ≤ 0.1. For example, Bedon *et al.* [16] used a double multiple streamtube momentum model without dynamic stall corrections to evaluate the effectiveness of various foil coefficient databases against the Sandia Darrieus turbine experimental data. Despite using such a simple model, performance predictions were quite accurate in most conditions except at low tip speed ratio for the 2 m turbine, which had the highest *c*/*R* of all the Sandia rotors, making the dynamic effects more important. This highlights the need for more validation data for higher solidity rotors to ensure numerical models are robust enough to explore unique cross-flow turbine designs, especially as the MHK concepts mature.

The UNH-RVAT, for which the performance, near-wake, and Reynolds number dependence were investigated using the same experimental setup as the study reported here [10, 32], was an H-rotor of 1 m height and 1 m diameter. The UNH-RVAT experimental datasets are also openly available [9, 30], and provide an interesting comparison for the near-wake dynamics of a rotor similar in size to the 1:6 scale RM2, but with non-tapered blades and a high solidity *c*/*R* = 0.28.

## Materials and Methods

### Turbine Model

**Fig 1:**
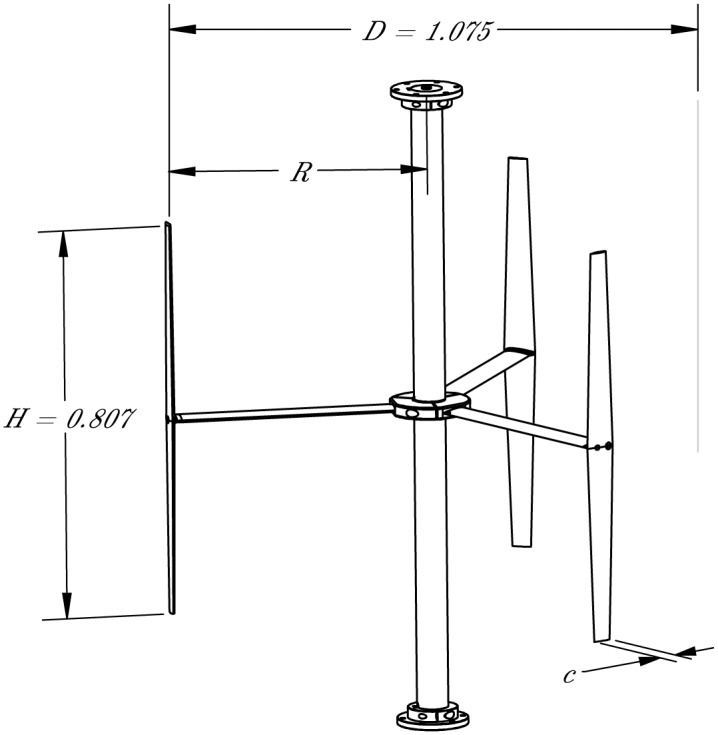
Turbine Model. A drawing of the RM2 turbine model. All dimensions are in meters.

Geometric parameters for the 1:6 scale RM2 rotor were taken from the RM2 design report [3], with the exception of the shaft diameter, which was scaled from the SAFL RM2 model [6]. Values for both the 1:6 and full-scale designs are presented in Table 2 and a drawing of the turbine is shown in Fig 1. The rotor components—blades, struts, shaft, and center hub sections—were fabricated from 6061-T6 aluminum, which was hardcoat anodized per MIL-8625-A, type III, class 2 specifications. CAD models of the turbine are available from [33].

**Table 2:** Turbine geometric parameters for both the full-scale and 1:6 scale model RM2 (tested here), along with the UNH-RVAT for comparison.

	Full-scale RM2	1:6 scale RM2	UNH-RVAT
Diameter (m)	6.450	1.075	1.0
Height (m)	4.840	0.8067	1.0
Blade root chord (m)	0.4000	0.06667	0.14
Blade tip chord (m)	0.2400	0.04000	0.14
Blade profile	NACA 0021	NACA 0021	NACA 0020
Blade mount	1/2 chord	1/2 chord	1/2 chord
Blade pitch (deg.)	0.0	0.0	0.0
Strut profile	NACA 0021	NACA 0021	NACA 0020
Strut chord (m)	0.3600	0.06000	0.14
Shaft diameter (m)	0.2540 [34] or 0.4160 [6]	0.06350	0.095

The rotor was 1.075 m in diameter and 0.8067 m tall, with blade chords that taper from 0.067 m at the roots, or half-span, to 0.04 m at the tips, varying the chord-to-radius ratio *c*/*R* from 0.12 to 0.07 (0.1 average). The rotor was therefore approximately at the threshold between high and low solidity [12, 35], which presents a unique validation case not yet seen in the literature. The RM2 is conceptually similar to the VAWT 850 [14] which also had tapered blades, but the RM2 has a moderately high *c*/*R* to more accurately represent typical MHK rotors, which presents a challenge for numerical modeling.

**Fig 2:**
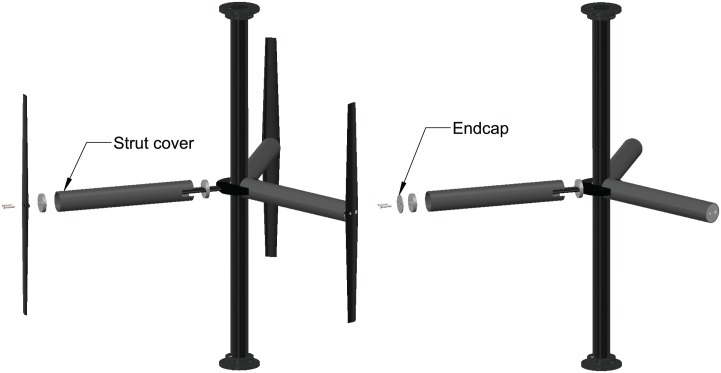
Strut Covers. A drawing of the high drag strut cover configuration with and without blades.

For investigating the effects of support strut drag losses, a set of cylindrical covers were designed to slip over the struts, which provided a deliberate and dramatic increase in strut drag. Endcaps were also fabricated to allow the high drag strut cover configuration to be operated without blades. A drawing of the strut covers is shown in Fig 2.

### Facility and instrumentation

**Fig 3:**
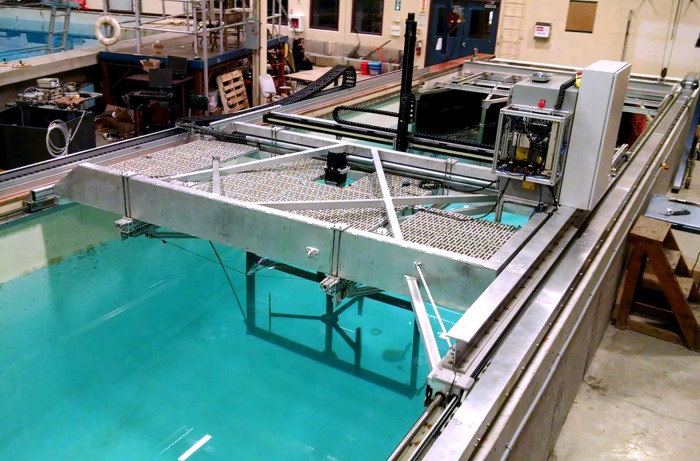
Experimental setup. Photo of the UNH tow tank and turbine test bed with RM2 installed.

**Fig 4:**
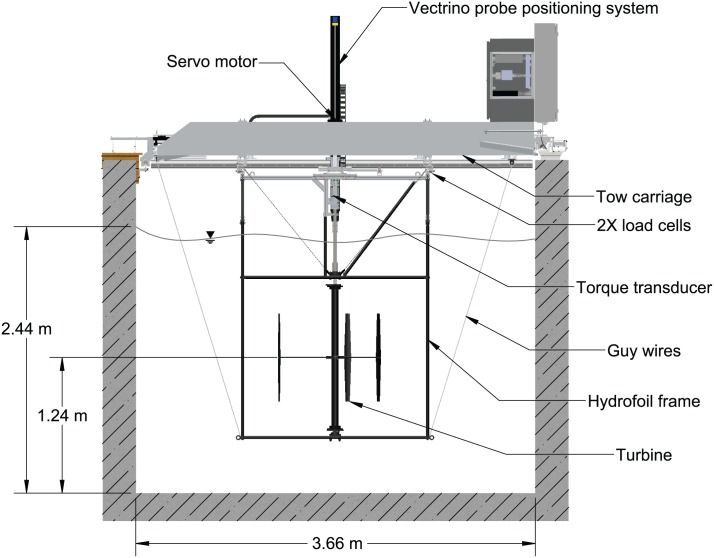
Experimental setup. Illustration of the experimental setup.

Measurements were conducted in the UNH tow/wave tank, pictured in Fig 3, a 36 m long facility with a 3.66 m wide by 2.44 m deep cross-section, capable of tow speeds up to 3 m/s. The blockage ratio based on the rotor frontal and tank cross-sectional area was 10%. The turbine was mounted in a frame built from NACA 0020 struts, attached to the tow carriage by four linear bearings, which transfer all streamwise force to a pair of 500 lbf capacity Sentran ZB3 S-beam load cells [36]. The turbine shaft RPM was controlled by a servo motor system, which allowed precise control of the turbine tip speed ratio. The load torque was measured by an Interface T8 200 Nm capacity inline rotary torque transducer [37]. An additional Sentran ZB3 load cell mounted at a fixed distance from the servo motor via a moment arm provided a redundant torque measurement, measurements from which were nearly identical to the inline transducer, providing additional confidence in the results. Turbine shaft angle was measured using the Kollmorgen AKD servo drive’s emulated encoder output [38], set to 10^5^ counts per turbine shaft revolution. Carriage speed, and therefore inflow velocity was measured using a Renishaw LM15 linear encoder with 10 *μ*m resolution [39]. All of these performance-related quantities were sampled at 2 kHz, while the tow tank’s motion controller provided redundant measurements of the carriage speed turbine angular velocity sampled at 1 kHz. Turbine wake measurements at 1 meter downstream were measured with a Nortek Vectrino+ acoustic Doppler velocimeter [40], sampling at 200 Hz. A photo and drawing of the experimental setup are shown in Figs 3 and 4, respectively.

#### Synchronization

The three data acquisition instrumentation subsystems—motion controller, NI DAQ (performance measurements), and Vectrino+ (wake velocity measurements)—started sampling at precisely the same time each tow, after being triggered by a digital signal edge sent from the motion controller. This strategy retained synchronization for all performance signal samples (tow speed, torque, drag, angular velocity), ensuring precise calculation of turbine performance parameters, e.g., power coefficient. Since there was also synchronization of the initial sample from each of the three subsystems, correlation of events in the performance and wake signals is also possible.

#### Tare drag and torque compensation

To best estimate the hydrodynamic loads on the turbine rotor alone, tare torque and drag runs were performed to measure the shaft bearing friction torque and turbine mounting frame drag, respectively. Tare drag runs—measured with the turbine removed—were performed for each tow speed in the experiment, for which the mean value was subtracted in data processing, to arrive at an estimate for drag on the turbine alone. Tare torque runs were performed by rotating the turbine shaft (without blades) in air at constant angular velocity for a specified duration, over the range of angular velocities used throughout the experiment. Tare torque was fit with a linear regression versus shaft angular velocity, and added to the measured turbine torque in post-processing.

### Test parameters

Data collection runs were separated into individual tows, for which all independent variables—tow speed, tip speed ratio, velocity probe position—were held constant. These runs were grouped into logical test matrix “sections,” in which typically a single independent variable was varied. Test matrix section names and descriptions are provided in the README.md file of the experimental data and code repository [11]. Tow speeds and their corresponding turbine diameter and blade chord Reynolds numbers are presented in Table 3.

**Table 3:** Test Reynolds numbers. Turbine diameter and approximate average blade chord Reynolds numbers *Re*_*c*_ ≡ λ*U*_∞_ *c*/*ν* at blade tip, root, and mid-span, corresponding to various tow speeds at λ = 3.1.

Tow speed (m/s)	*Re*_*D*_	*Re*_*c*_*tip*__	*Re*_*c*_*root*__	*Re*_*c*_*mid*__
0.4	4.3 × 10^5^	5.0 × 10^4^	8.3 × 10^4^	6.6 × 10^4^
0.6	6.5 × 10^5^	7.4 × 10^4^	1.2 × 10^5^	9.9 × 10^4^
0.8	8.6 × 10^5^	9.9 × 10^4^	1.7 × 10^5^	1.3 × 10^5^
1.0	1.1 × 10^6^	1.2 × 10^5^	2.1 × 10^5^	1.7 × 10^5^
1.2	1.3 × 10^6^	1.5 × 10^5^	2.5 × 10^5^	2.0 × 10^5^

**Fig 5:**
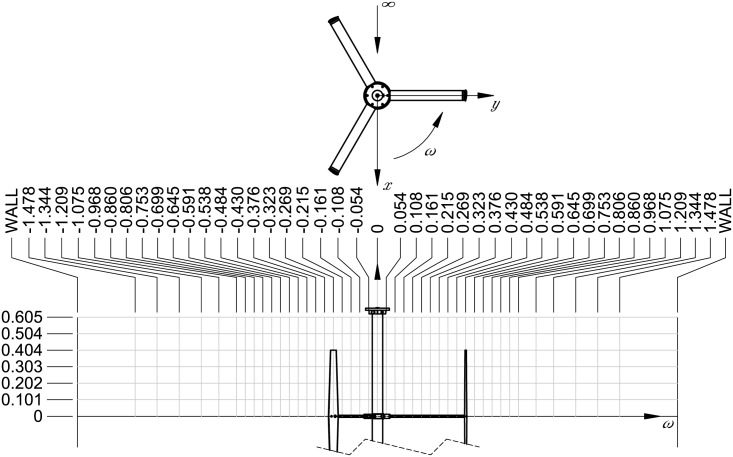
Coordinate system. Wake measurement coordinate system and cross-stream/vertical coordinates. All dimensions are in meters.

Wake measurements were all performed at 1 m downstream, which corresponds to *x*/*D* = 0.93. The cross-stream and vertical coordinates are shown in Fig 5. Altogether 750 tows were performed and included in the experimental database.

### Uncertainty Analysis

For the mean rotor power and drag coefficients an expanded uncertainty interval with 95% confidence

$$\begin{array}{c} U_{95}=t_{95}u_{c}, \end{array}$$ (1)

was computed, where *t*_95_ is the value from the Student-*t* distribution for a 95% confidence interval and *u*_*c*_ is the combined standard uncertainty [41]. Combined standard uncertainty for the sample mean of a given quantity *X* is calculated by

$$\begin{array}{c} u_{X}^{2}=s_{\bar{X}}^{2}+b_{X}^{2}, \end{array}$$ (2)

where $s_{\bar{X}}$ is the sample standard deviation of the mean per turbine revolution ($s_{\bar{X}}=s_{X}/\sqrt{N}$), and *b*_*X*_ is the systematic uncertainty, computed as

$$\begin{array}{c} b_{X}^{2}=\sum_{i=1}^{J}{\left(\frac{\partial X}{\partial x_{i}}\right)}^{2}b_{x_{i}}^{2}, \end{array}$$ (3)

where *x*_*i*_ is a primitive quantity used to calculate *X* (e.g. *T*, *ω*, and *U*_∞_ for calculating *C*_*P*_), and *b*_*x*_*i*__ is the primitive quantity’s systematic uncertainty, estimated as half the value listed on the sensor manufacturer’s calibration or datasheet.

Selecting *t*_95_ requires an estimate for degrees of freedom *ν*_*X*_, which was obtained using the Welch–Satterthwaite formula

$$\begin{array}{c} \nu_{X}=\frac{{\left(s_{X}^{2}+\sum_{k=1}^{M}b_{k}^{2}\right)}^{2}}{s_{X}^{4}/\nu_{s_{X}}+\sum_{k=1}^{M}b_{k}^{4}/\nu_{b_{k}}}, \end{array}$$ (4)

where *ν*_*s*_*X*__ is the number of degrees of freedom associated with *s*_*X*_ and *ν*_*b*_*k*__ is the number of degrees of freedom associated with *b*_*k*_. *ν*_*s*_*X*__ is assumed to be (*N* − 1), where *N* is the number of independent samples (or turbine revolutions). The degrees of freedom parameter *ν*_*b*_*k*__ was estimated as

$$\begin{array}{c} \nu_{b_{k}}=\frac{1}{2}{\left(\frac{\Delta b_{k}}{b_{k}}\right)}^{-2}, \end{array}$$ (5)

where the quantity in parentheses is the relative uncertainty of *b*_*k*_, assumed to be 0.25.

### Data Processing

For each tow speed, a relevant quasi-steady duration was selected by manually inspecting a plot of the *C*_*P*_ time series. This interval was then truncated to include a whole number of blade passages. Relevant statistics were then calculated over this duration.

Since the blockage ratio was relatively low (10%), and a definitive blockage correction method for cross-flow turbines has not been established [42] blockage effects were not corrected for. Furthermore, most numerical modeling efforts can easily model finite domains, i.e., incorporate tow tank walls. Therefore, applying blockage corrections to experimental data could actually complicate validation efforts.

To calculate turbine RPM from shaft angle, the encoder signal was differentiated using a second order central difference scheme, after which an 8 sample wide moving average smoothing filter was applied to match the noise level present in the redundant turbine RPM measurement from the motion controller. A similar approach was used for calculating tow carriage speed *U*_∞_ from carriage position measurements. Power and drag coefficients are calculated as instantaneous quantities from the carriage speed as

$$\begin{array}{c} C_{P}=\frac{T\omega }{\frac{1}{2}\rho AU_{\infty }^{3}} \end{array}$$ (6)

and

$$\begin{array}{c} C_{D}=\frac{F_{\text{drag}}}{\frac{1}{2}\rho AU_{\infty }^{2}}, \end{array}$$ (7)

where *ρ* is the fluid density (assumed to be a nominal 1000 kg/m^3^) and *A* is the turbine frontal area *DH*.

All data processing and plotting code, along with the reduced dataset, are available from [11]. Note that this code will automatically download raw data as necessary so users can perform a full reanalysis of the measurements presented here.

## Results and Discussion

### Performance

**Fig 6:**
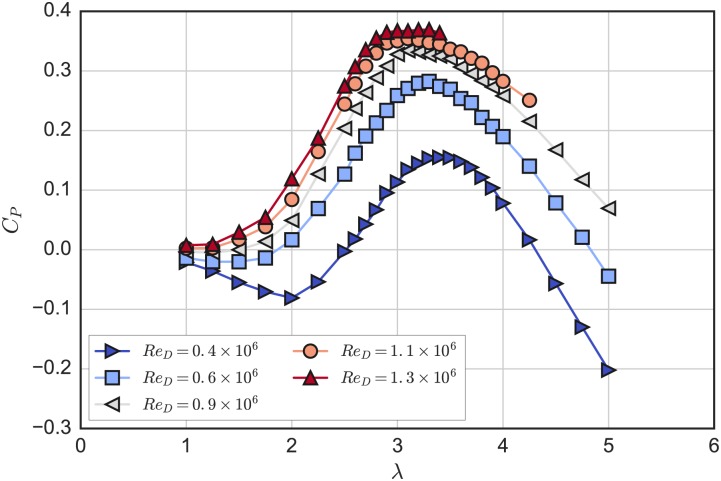
Power Coefficient Curves. Mean rotor power coefficient plotted versus mean tip speed ratio for multiple Reynolds numbers.

Mean rotor power coefficients for multiple Reynolds numbers are plotted versus tip speed ratio in Fig 6. In general, *C*_*P*_ increases with *Re*, along with a reduction in the optimal tip speed ratio, due to the tendency of foils to stall at higher angles of attack at higher *Re*, and the higher angle of attack ranges seen by the blades at lower λ. These effects diminish with increasing *Re*, which is expected as the blade boundary layers transition to turbulence closer to the leading edge [9, 43, 44], which helps flow remain attached longer as it moves against the adverse pressure gradient on the suction side of the foil. For the experiment reported here, a maximum power coefficient *C*_*P*_ = 0.37 was reached at *Re*_*D*_ = 1.3 × 10^6^. Note how at lower tip speed ratios and Reynolds numbers, *C*_*P*_ becomes significantly negative, which was possible with the speed control of the experimental setup’s servo motor applying negative torque to the rotor.

**Fig 7:**
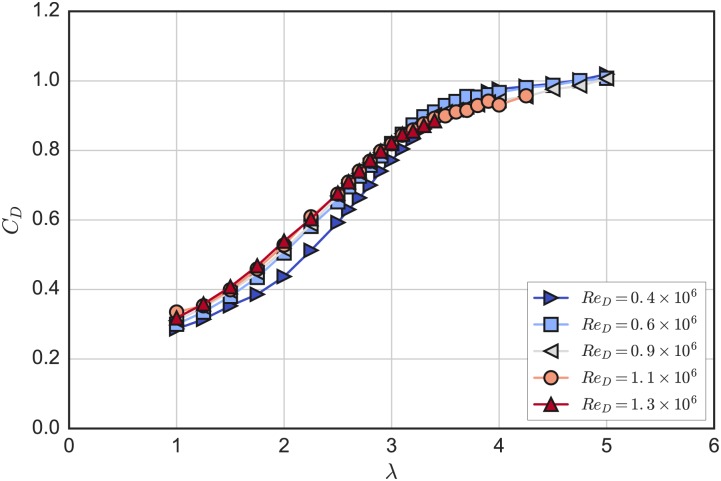
Drag Coefficient Curves. Mean rotor drag coefficient plotted versus mean tip speed ratio for multiple Reynolds numbers.

Mean rotor drag (or thrust) coefficients are plotted versus tip speed ratio in Fig 7. These *C*_*D*_ curves show little difference compared to the power coefficient curves; differences are most noticeable at low λ and low *Re*. The relative similarity could be attributed to the effects of stall, where the lift-to-drag ratio on the blades may drop, decreasing rotor torque, though the total resultant force due to high blade drag retains a similar component in the streamwise direction. When operating at maximum power coefficient, λ_0_ = 3.1, and *Re*_*D*_ = 1.3 × 10^6^, a rotor drag coefficient *C*_*D*_ = 0.84 was measured.

The power coefficient curves do not collapse exactly onto each other, indicating Reynolds number dependence, though the differences become relatively small as *Re* is increased. Note that the data collection was limited at higher *Re* and λ due to the unsteady turbine force resonating with the tow carriage drive belt. However, in contrast to the power coefficient data, the rotor drag coefficient curves nearly collapse onto each other for *Re*_*D*_ ≥ 0.6 × 10^6^.

**Fig 8:**
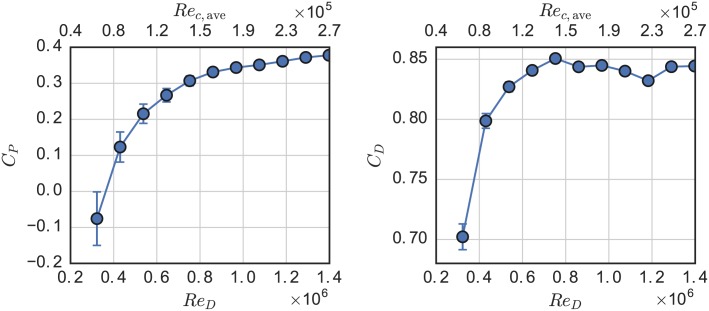
RM2 Reynolds Number effects on performance. Power (left) and drag (right) coefficient at λ = 3.1 plotted versus turbine diameter and approximate average blade root chord Reynolds number.

The effects of Reynolds number on the power and drag coefficients at λ = 3.1 are shown in Fig 8. The rotor drag coefficient *C*_*D*_ became more or less Reynolds number independent for *Re*_*D*_ ≥ 0.6 × 10^6^. The power coefficient of the turbine increased dramatically below *Re*_*D*_ = 1 × 10^6^ or *Re*_*c*_ ≡ λ*U*_∞_ *c*/*ν* ≈ 2 × 10^5^, beyond which there appears to be a small, linear, positive trend. At the lowest Reynolds number, mean power coefficient even dropped below zero, which is consistent with the low performance of the 1:15 scale RM2 physical model study [6]. However, the blades of the 1:15 scale RM2 were mounted at approximately 58% of the chord from the leading edge, versus 50% for the present model, which helps explain the 1:15 scale model’s positive power output at λ = 2.2 and *Re*_*D*_ ∼ 10^5^, since moving the blade mount point further back is equivalent to a “toe-out” preset pitch condition [35].

**Fig 9:**
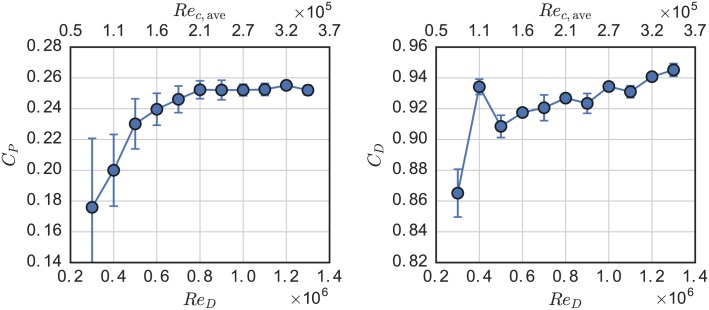
UNH-RVAT Reynolds Number effects on performance. Power (left) and drag (right) coefficient at λ = 1.9 plotted versus turbine diameter and approximate average blade chord Reynolds number. Taken from [32].

The tendency of *C*_*P*_ to continue increasing slightly could be an effect of flow curvature—caused by the finite *c*/*R*—which imparts a “virtual camber” [45], or can be thought of as producing a “lead” in angle of attack since cambered airfoils have non-zero lift at zero angle of attack [46]. The residual weak *Re*-dependence of the RM2 compared to the *Re*-independence of the higher *c*/*R* UNH-RVAT, shown in Fig 9, could be due to this virtual camber effect, since camber has been shown to cause earlier *Re*-convergence of the CFT’s geometric torque coefficient when calculated from static foil coefficients given by a viscous panel method, whereas characteristics like lift-to-drag ratio do not converge as strongly [32].

### Strut drag losses

**Fig 10:**
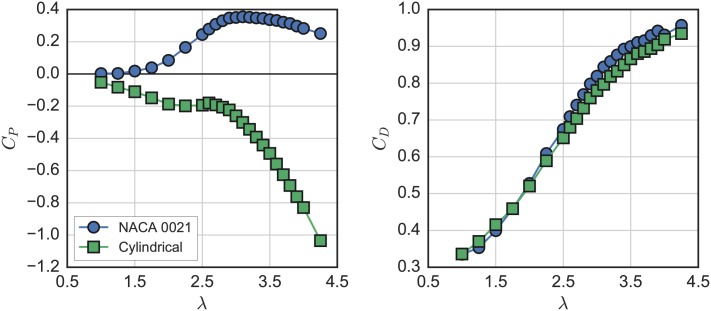
High drag strut performance. Turbine performance and rotor drag coefficient curves with both NACA 0021 and cylindrical struts.

Performance curves for the rotor with both NACA 0021 and cylindrical struts are shown in Fig 10. As expected, the high drag cylindrical struts reduce performance dramatically, producing an entirely negative *C*_*P*_ curve. The detrimental effects of the strut drag were more pronounced at higher λ. This is in accordance with the measurements of Rawlings [47], though the strut losses in this case were larger due to the very high drag circular profile. In contrast to the dramatic effect strut drag had on *C*_*P*_, overall rotor drag measurements were relatively similar for both the cylindrical and NACA 0021 strut cases.

**Fig 11:**
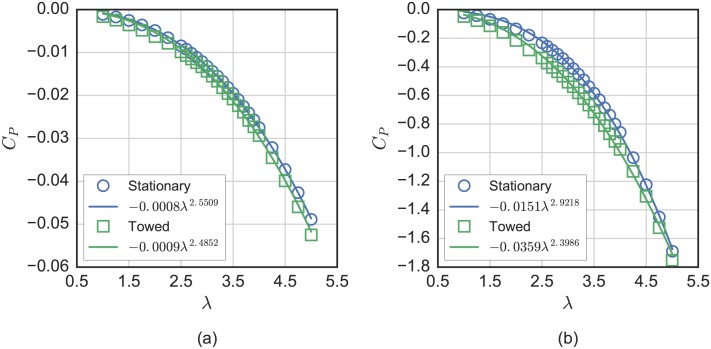
Strut drag losses. Measurements of the strut drag losses for (a) NACA 0021 and (b) cylindrical struts, both stationary and towed at 1 m/s. Note that stationary calculations for tip speed ratio and power coefficient also assumed *U*_∞_ = 1 m/s.

Measurements for the power coefficient contributions of the strut drag losses are presented in Fig 11 for NACA 0021 and cylindrical struts—both in towed (at *U*_∞_ = 1 m/s) and stationary conditions. These are computed in the same fashion as the curves in Fig 6, but with the rotor blades removed. For the stationary case, tip speed ratio and power coefficient were computed using the same reference velocity *U*_∞_ = 1 m/s. We see that strut drag losses increase with tip speed ratio to the power 2–3, which makes streamlined struts much more important for low solidity turbines, given the inverse correlation between solidity and λ_0_ [48].

Strut drag losses did not change much for the streamlined NACA 0021 struts in the towed versus stationary configuration, which helps explain why overall rotor drag coefficients shown in Fig 10 remained of comparable magnitude even at low Reynolds number, where *C*_*P*_ was dramatically reduced, or even negative. For the cylindrical struts, losses increased significantly when towed and operating in the mid range of tip speed ratios.

Though a real turbine would never use such high drag struts, with respect to numerical modeling, these measurements provide some interesting validation cases. Modelers can isolate and evaluate the ability to predict these losses independent of the blade loading by modeling combinations of the rotor with the high/low drag struts and with/without blades.

### Near-wake characteristics

**Fig 12:**
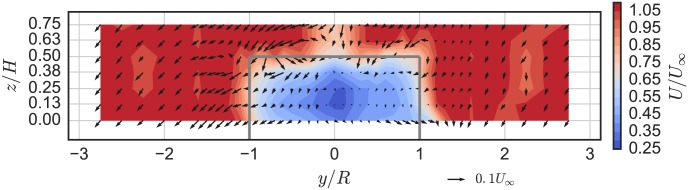
RM2 Near-Wake Mean Velocity. RM2 near-wake mean velocity field (looking upstream) at 1 m downstream (*x*/*D* = 0.93), *U*_∞_ = 1.0 m/s, and λ = 3.1. Refer to Fig 5 for turbine axis orientation. Solid dark gray lines indicate turbine frontal area.

**Fig 13:**
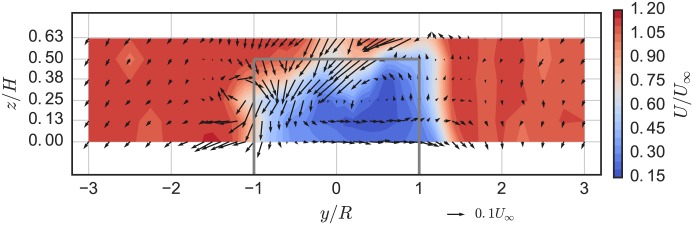
UNH-RVAT Near-Wake Mean Velocity. UNH-RVAT near-wake mean velocity field (looking upstream) at 1 m downstream (*x*/*D* = 1.0), *U*_∞_ = 1.0 m/s, and λ = 1.9, from [32]. Solid dark gray lines indicate turbine frontal area.

The mean velocity field at the chosen optimal tip speed ratio λ_0_ = 3.1, 1 m downstream (*x*/*D* = 0.93) from the rotor axis is plotted in Fig 12. The mean streamwise velocity deficit is markedly more symmetric than that of the higher solidity UNH-RVAT, shown in Fig 13, with the RM2 inducing lower acceleration around the turbine due to the lower rotor drag coefficient and a slightly lower blockage ratio [10]. Tip vortex shedding is relatively weaker, which is likely an effect of the RM2’s smaller blade chord length and tapered blades.

**Fig 14:**
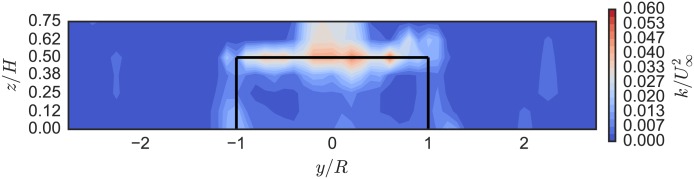
RM2 Near-Wake Turbulence Kinetic Energy. Turbulence kinetic energy in the RM2’s near-wake (looking upstream) at 1 m downstream (*x*/*D* = 0.93), *U*_∞_ = 1.0 m/s, and λ = 3.1. Solid black lines indicate turbine frontal area.

**Fig 15:**
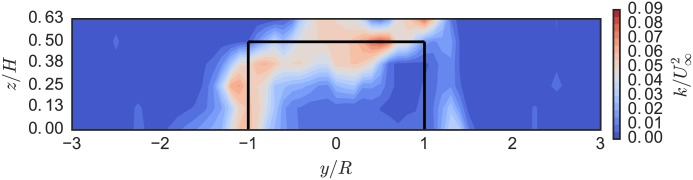
UNH-RVAT Near-Wake Turbulence Kinetic Energy. Turbulence kinetic energy in the UNH-RVAT’s near-wake (looking upstream) at 1 m downstream (*x*/*D* = 1.0), *U*_∞_ = 1.0 m/s, and λ = 1.9, from [32]. Solid black lines indicate turbine frontal area.

Fig 14 shows the turbulence kinetic energy in the turbine wake. We mainly see unsteadiness in the flow generated by the blade tip vortex shedding (the horizontal band around *z*/*H* = 0.5). Compared with the UNH-RVAT, shown in Fig 15, turbulence generation is lower overall, without the intense vertical band around *y*/*R* = −1, which indicates that the RM2 blades are operating further from stall—consistent with its higher optimal tip speed ratio.

Analysis of the near-wake of the higher solidity UNH-RVAT turbine revealed that vertical advection was the largest contributor to streamwise recovery [10], a trait which is considered an advantage of cross-flow over axial-flow rotors in arrays [49]. A similar analysis was undertaken for the RM2, starting with the transport equation for mean kinetic energy, rearranged to isolate the streamwise partial derivative (indices follow the Einstein summation convention):

$$\begin{array}{ccc} \frac{\partial K}{\partial x} & = & \frac{1}{U}[-\underset{y\text{-adv.}}{\underset{︸}{V\frac{\partial K}{\partial y}}}-\underset{z\text{-adv.}}{\underset{︸}{W\frac{\partial K}{\partial z}}}-\frac{1}{\rho }\frac{\partial }{\partial x_{j}}PU_{i}\delta_{ij}+\frac{\partial }{\partial x_{j}}2\nu U_{i}S_{ij}-\underset{\text{Turb.}\;\text{trans.}}{\underset{︸}{\frac{1}{2}\frac{\partial }{\partial x_{j}}\bar{u_{i}^{\prime }u_{j}^{\prime }}U_{i}}}\\ & & +\;\underset{k\text{-prod.}}{\underset{︸}{\bar{u_{i}^{\prime }u_{j}^{\prime }}\frac{\partial U_{i}}{\partial x_{j}}}}-\underset{\text{Mean}\;\text{diss.}}{\underset{︸}{2\nu S_{ij}S_{ij}}}]. \end{array}$$ (8)

Terms that were able to be calculated from the experimental data are those that do not involve *x*-derivatives, since all wake measurement locations were at a fixed downstream distance. The available terms are the cross-stream advection (*y*-adv.), vertical advection (*z*-adv.), transport due to turbulent fluctuations (*y*-turb. and *z*-turb., separated by the direction of the derivative), production of turbulence kinetic energy (*k*-prod.), and the dissipation due to the mean velocity gradient (Mean diss.).

**Fig 16:**
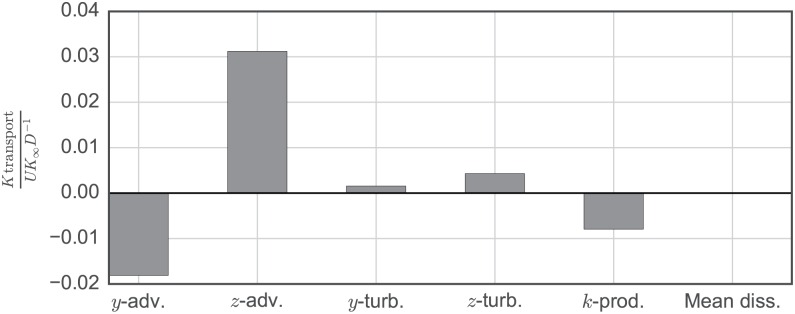
Mean Kinetic Energy Transport. Weighted average estimates for terms contributing to streamwise recovery of mean kinetic energy, multiplied by two due to implied symmetry.

Derivatives were computed with a second order central difference scheme for interior points, and a second order inward-facing scheme for the edges, following the methodology in [10]. Weighted averages for these calculations are shown in Fig 16. Due to the weaker blade vortex shedding, transport due to vertical advection at this point in the wake was approximately 3 times lower than the higher solidity UNH-RVAT. Note that direct comparison is somewhat invalid, since the total measurement plane area was about 5% lower for this experiment. However, the differences observed are larger than the differences in measurement plane area. We also see relatively lower levels of cross-stream turbulent transport due to the lack of blade stall vortex shedding. These results may have interesting implications regarding the application of turbines with lower power coefficient to possibly improve overall array performance through enhanced transport of kinetic energy from the free stream, though evaluating these trade-offs will require a detailed analysis of the downstream evolution and turbine–wake interaction.

## Conclusions

The performance and near-wake velocity of a 1:6 scale DOE Reference Model 2 cross-flow turbine were measured in a towing tank. A maximum power coefficient *C*_*P*_ = 0.37 and rotor drag coefficient *C*_*D*_ = 0.84 were observed at an optimal tip speed ratio λ_0_ = 3.1.

Performance was assessed for Reynolds number dependence, showing a convergence to a weakly *Re*-dependent linear regime at approximately *Re*_*D*_ ≈ 1 × 10^6^ or *Re*_*c*, *ave*_ ≈ 2 × 10^5^. Comparison was made between this turbine and the higher solidity UNH-RVAT, tested in nearly identical conditions, which showed similar Reynolds number thresholds but a flatter linear regime, likely due to virtual camber caused by the higher chord-to-radius ratio of the UNH-RVAT blades. Nevertheless, these results indicate an important transitional scale threshold beyond which data should be taken for numerical model validation, in order to stay relevant to full scale devices.

The effects of parasitic drag from blade support struts on turbine performance were measured by rotating the turbine without blades while stationary and while towing. These losses—even for a streamlined hydrofoil strut—can become significant at higher tip speed ratios—up to an approximate 5 percentage point decrease in power coefficient at a tip speed ratio of 5. These measurements were repeated with a set of high-drag cylindrical struts, which as expected, prevented the turbine from producing any mechanical power at any tip speed ratio. Nevertheless these measurements provide useful validation data for both high and low fidelity numerical performance prediction models, allowing researchers and engineers to test predictions without blade effects.

While operating at its optimal tip speed ratio λ = 3.1 the wake at *x*/*D* = 0.93 downstream was shown to be relatively symmetrical and lacked the evidence of strong blade stall, both of which differentiate this near-wake from the higher solidity, lower λ_0_ UNH-RVAT. Terms from the mean kinetic energy transport equation were also computed in this *y*–*z* plane, showing the relative importance of the vertical advection compared with turbulent transport terms at this location, which is qualitatively similar to the UNH-RVAT wake data. However, lower wake recovery totals were calculated. This indicates that although the RM2 is a more effective energy converter than the UNH-RVAT, its wake recovery may in fact be delayed due to weaker blade tip vortex shedding and lower levels of turbulence in the near-wake, which may present a trade-off when considering optimal array layouts for maximizing power output per unit footprint area.
